# A Study on Central Lymph Node Metastasis in 543 cN0 Papillary Thyroid Carcinoma Patients

**DOI:** 10.1155/2016/1878194

**Published:** 2016-04-03

**Authors:** Huanhuan Yan, Xiaoqian Zhou, Hui Jin, Xiang Li, Miao Zheng, Xu Ming, Ruitao Wang, Jun Liu

**Affiliations:** Department of Breast-Thyroid-Vascular Surgery, Shanghai General Hospital of Nanjing Medical University, 201620 Shanghai, China

## Abstract

*Background*. Papillary thyroid carcinoma (PTC) with central lymph node metastases (CLNMs) is common. The objective of this study was to investigate the incidence and risk factors of lymph node metastasis patients with PTC.* Patients and Methods*. Between January 2013 and February 2015, a retrospective study of 543 patients with PTC undergoing hemithyroidectomy or total thyroidectomy with routine central lymph node dissection (CLND) was analyzed. Clinicopathologic risk factors for CLNM were studied using univariate and multivariate analysis by SPSS 22.0 software.* Results*. The incidence of CLNMs in PTC patients was 38.1% (207/543). In the multivariate analysis, male gender (*p* < 0.001, OR: 1.984), age <45 years (*p* < 0.001, OR: 1.934), bilaterality (*p* = 0.006, OR: 1.585), tumor size ≥0.25 cm (*p* = 0.001, OR: 7.655), and external extension (*p* = 0.001, OR: 7.579) were independent risk factors of CLNMs. Furthermore, in PTC patients with tumor size <0.25 cm, all 7 males and 21 patients with unilaterality were not found to have CLNMs.* Conclusions*. CLNMs are prevalent in the PTC patients with the following risk factors: male gender, age <45 years, bilaterality, tumor size ≥0.25 cm, and external extension. PTC patients with tumor size <0.25 cm, male patients, and patients with unilateral lesion could be considered safe from CLNMs.

## 1. Introduction

Papillary thyroid carcinoma (PTC) is the most common form of thyroid gland carcinoma which accounts for 1% of all human malignant tumors. It was reported that the prognosis of PTC is excellent with a 10-year survival exceeding 90% [1]. However, approximately 20–50% of PTC patients were found to have central lymph node metastases (CLNMs) [2, 3]. There is still a recurrence rate of 8–25% after primary surgical treatment [4], and many scholars hold the opinions that routine central lymph node dissection (CLND) may decrease the rate of recurrence.

## 2. Patients and Methods

This retrospective study was approved by Department of Breast-Thyroid-Vascular Surgery at Shanghai General Hospital of Nanjing Medical University. From January 2013 to February 2015, a total of 543 PTC patients who underwent radical operation of thyroid were enrolled in this study. Total thyroidectomy and bilateral CLND were considered when the cN0 patients had bilateral PTC. Total thyroidectomy and ipsilateral CLND might be performed for unilateral PTC patients who met one or more of the following conditions according to the NCCN guidelines: radiation history, known distance metastases, extrathyroidal extension, and tumor size >4 cm in diameter. Other unilateral PTC patients underwent unilateral and contralateral partial resection of thyroid gland or hemithyroidectomy with ipsilateral CLND. The CLN was group VI lymph nodes. Its upper bound was to the thyroid cartilage, and its lower bound was to thymus, and both sides were to the inner side of the central bilateral carotid sheath, including lymph nodes prior to cricothyroid membrane, lymph nodes surrounding thyroid, and paratracheal and prelaryngeal lymph node. Intraoperative frozen section (FS) is a popular method for doctors to determine surgical extent in surgery. It was reported that the accuracy rates for FS in thyroid surgery was 97.8% [5]. And intraoperative FS was performed in all patients. Preoperative ultrasonography, preoperative thyroid function tests, and postoperative pathologic examination were performed in all patients.

Based on the NCCN guidelines for PTC [6], all of the 543 patients with clinically lymph node that was negative according to the preoperative physical examination and ultrasound underwent the surgical treatment. Total thyroidectomy with bilateral/unilateral CLND was performed in 419 patients. 79 patients underwent unilateral and contralateral partial resection of thyroid gland with bilateral/unilateral CLND. Hemithyroidectomy with bilateral/unilateral CLND was performed in 39 patients. Six patients performed secondary surgical intervention because they were diagnosed as having benign lesions according to intraoperative FS, but the postoperative pathological diagnosis of the first surgery was PTC. In this study, the preoperative fine needle aspiration was carried out on 337 patients, and 322 cases were diagnosed with PTC. From a total of 543 patients, 537 cases were diagnosed with PTC by the intraoperative FS, and 6 cases were diagnosed postoperatively after the histopathological examination of thyroid gland. LT4 was given to all the patients as an alternative treatment besides the inhibition of TSH therapy.

## 3. Statistics Analysis

Chi-square test or Fisher's exact test was used to analyze the significance of differences of the categorical variables and logistic regression was performed to analyze the multiplicity. All the data were processed using Statistic Package for Social Science (SPSS 22.0) and *p* < 0.05 was considered as a statistically significant difference.

## 4. Results

### 4.1. Clinical and Pathological Characteristics

410 (75.5%) females and 133 (24.5%) males were included in this study. The average age of all the patients was 47 years, ranging from 16 to 78 years. The mean size of the tumors was 0.95 ± 0.73 cm (ranging from 0.1 cm to 5 cm). 132 (24.3%) patients had bilateral lesions, 180 (33.1%) cases had multifocal lesions, 517 (95.2%) cases had tumor ≥0.25 cm in size, and 36 (6.6%) cases had PTC with extrathyroidal extension. 190 (35.0%) cases had PTC with Hashimoto's thyroiditis (HT) and 78 (14.4%) cases had abnormal thyroid function.

### 4.2. Risk Factors for PTC with CLNMs

We analyzed these factors including the gender, age, bilaterality, multifocality, tumor size, extrathyroidal extension, HT, and abnormal thyroid function to find the risk factors for PTC patients with CLNMs. In the univariate analysis, male gender (*p* = 0.012), age <45 years (*p* < 0.001), bilateral lesions (*p* = 0.042), tumor size ≥0.25 cm (*p* = 0.003), and external invasion (*p* = 0.003) were significantly associated with CLNMs. Multifocal lesions, HT, and abnormal thyroid function were not significant for CLNMs (Table 1).

The multivariate analysis (Table 2) showed that the male gender (*p* < 0.001, OR: 1.984), age <45 years (*p* < 0.001, OR: 1.934), bilateral lesions (*p* = 0.006, OR: 1.585), tumor size ≥0.25 cm (*p* = 0.001, OR: 7.579), and external invasion (*p* = 0.002, OR: 2.370) were independent risk factors for CLNMs in PTC patients.

We grouped all the PTC patients into five groups based on the size of tumors: <0.25 cm, ≥0.25 and <0.5 cm, ≥0.5 and <0.75 cm, ≥0.75 and <1 cm, ≥1 cm. And the rate of CLNMs increased as the tumor size increased. There were significant differences for these groups (*p* < 0.001) (Table 3).

Among the 543 cN0 PTC patients, 38.1% (207/543) were found to pathologically have CLNMs.  Table 4 showed that no CLNMs were found in all 7 males and 21 patients with unilateral lesion in cN0 PTC patients with tumor size <0.25 cm. The percentages of patients with CLNMs whose conditions met only zero, one, two, three, four, or all of the five risk factors were 0 (0/6), 22.5% (39/173), 43.7% (104/238), 46.3% (50/108), 77.8% (14/18), and 0. Among the 6 patients without these five risk factors, 0% (0/6) were found to have CLNMs (Table 4).

## 5. Discussion

Papillary thyroid carcinoma which is considered to have relatively good prognosis still has at least 10% risk of recurrence in long-term follow-up [7–9]. CLNMs are the most important variable known to increase the risk of local recurrence [10]. A large study found that the mortality of PTC patients with CLNMs was much higher than that of patients without CLNMs [11]. Pellegriti et al. [12] kept the option that the development of distant metastases was associated with the presence of lymph node metastases at presentation. The role of routine CLND for cN0 PTC remains controversial. More and more scholars recommend that CLND is necessary for PTC patients because of the greater rate of CLNMs. Wang et al. [10] reported that 44.1% cN0 PTC patients were found to have CLNMs. Jiang et al. [13] reported that nearly 53.71% cN0 PTC patients had CLNMs. However, Machens et al. [14] considered that CLNMs were associated with local recurrence and distant metastasis but did not impair survival. Therefore, it is essential to investigate the indications for cN0 PTC patients.

Obviously, male gender, age <45 years old, bilaterality, tumor size ≥0.25 cm, and external invasion were independent risk factors for cN0 PTC patients. Several studies demonstrated that male PTC patients have higher significant risk of CLNMs [15, 16]. However, Jiang et al. [13] found that the gender had no association with CLNMs. In our study, the proportion of the male gender with CLNMs was significantly higher than that of female gender (47.4% versus 35.1%, *p* < 0.001, OR: 1.984). Age is often used to judge the stage of the differentiated thyroid carcinoma. We found that younger PTC patients (<45 years old) were at higher risk of occurring CLNMs (46.6% versus 23.9%, *p* < 0.001, OR: 1.934). Ahn et al. [17] analyzed 916 cN0 PTC patients and had similar finding that the rate of CLNMs was considerably higher in the cases of younger patients (*p* < 0.001; OR: 2.357). Jiang et al. [13] believed that age ≥35 years was a good prognostic factor for PTC patients with CLNMs. In agreement with Pellegriti et al. [12] and Vasileiadis et al. [18], we found that bilateral lesions made a meaningful difference with a higher percentage to develop CLNMs from the unilaterality (45.5% versus 35.7%, *p* = 0.006, OR: 1.585). It was accepted that the tumor size is associated with lymph node metastasis. Chang et al. [15] and Zhang et al. [19] have demonstrated that larger tumor size (>0.5 cm, >0.6 cm) was associated with CLNMs. In our study, PTC patients with tumor size ≥0.25 cm were more prone to develop CLNMs (39.7% versus 7.7%, *p* = 0.001, OR: 7.579). Many studies have demonstrated that external invasion is a risk factor of CLNMs for PTC patients [4, 13, 15, 18]. Our study showed that nearly 61.1% percent of PTC patients with external invasion was found to have CLNMs and the multivariate analysis indicated that external invasion was an independent risk factor for CLNMs (61.1% versus 36.5%, *p* = 0.002, OR: 2.370).

With the development of ultrasound, palpation, fine needle aspiration (FNA), and intraoperative frozen section, increasing small malignant thyroid nodules were checked out. Before the dispute regarding [20, 21] the operation for PTC patients with tumor size ≤1 cm, studies shall need to advance. In this study, the rate of tumor size with CLNMs increased, obviously, with the increase of tumor size in a certain range (<0.25 cm: 7.6%, ≥0.25 and <0.5 cm: 22.7%, ≥0.5 and <0.75 cm: 30.1%, ≥0.75 and <1 cm: 45.5%, ≥1 cm: 53.4%). 7.6% of PTC patients with tumor size <0.25 cm were confirmed to have metastases in central lymph node, while about 39.7% of PTC patients whose tumor size was ≥0.25 cm were confirmed to have CLNMs. The incidence of CLNMs in PTC patients with tumor size ≥0.25 and <0.5 cm was 22.7%. And the incidence of CLNMs in PTC patients with tumor size <0.5 cm was reported to be 20.6–26.2% [13, 15, 17]. Therefore, we think that CLND should be performed in PTC patients with tumor size ≥0.25 cm.

In this study, the incidence of CLNMs in PTC patients was 38.1%. Similar studies in cN0 PTC patients reported that the incidence of CLNMs was 35.0–43.6% [15, 16, 22]. The clinic risk factors for PTC patients with CLNMs were analyzed above, and we also evaluated the incidence of CLNMs with every conceivable combination to further analyze the indication of CLND. Eliminating tumor size ≥0.25 cm, no CLNMs were found in male patients and patients with unilateral lesion. In the present studies, CLND is necessary to be performed in the PTC patients because CLNMs are associated with a higher risk of recurrence [23, 24]. Hence, CLND should be performed in the patients with risk factors, and we recommend that, in cN0 PTC patients with tumor size <0.25 cm, male patients and patients with unilateral lesion should be considered safe.

In this study, some limitations should be considered. First of all, this retrospective study was short of the prognosis information. Secondly, all patients are required to have long-term follow-up to analyze the significance of CLND for cN0 PTC patients.

## 6. Conclusions

In summary, the incidence of PTC patients with CLNMs is 38.1% in our study. Male gender, age <45 years, bilaterality, tumor size ≥0.25 cm, and external extension were considered as independent risk factors for PTC patients. On this account, we think precautionary CLND should be considered for cN0 PTC patients with risk factors. And we believe that, in cN0 PTC patients with tumor size <0.25 cm, male patients and patients with unilateral lesion could avoid CLND.

## Figures and Tables

**Table 1:** Univariate analysis for PTC patients with CLND.

Risk factors	CLN		Univariate analysis	
	Positive	Negative	*χ* ^2^	*p*
Gender			6.385	0.012
Female	144	266		
Male	63	70		
Age (years)			11.2	0.001
<45	102	117		
≥45	105	335		
Bilaterality			4.131	0.042
Yes	61	73		
No	146	263		
Multifocality			3.797	0.051
Yes	79	101		
No	128	235		
Tumor size (cm)			9.407	0.002
<0.25	2	24		
≥0.25	205	312		
Extrathyroidal extension			8.639	0.003
Yes	22	14		
No	185	322		
HT			2.44	0.118
Yes	64	126		
No	143	210		
Abnormal thyroid function			0.102	0.75
Yes	31	47		
No	176	289		

**Table 2:** Multivariate analysis for risk factors of central lymph node metastasis.

Variables	SE	Wald	*p* value	Exp(*b*)	95% CI of Exp(*b*)	
Gender (male versus female)	0.169	16.544	0.000	1.984	1.427	2.762
Age (<45 versus 45 years)	0.165	15.864	0.000	1.934	1.397	2.674
Bilaterality (yes versus no)	0.167	7.655	0.006	1.585	1.144	2.198
Tumor size (<0.25 versus ≥0.25 cm)	0.608	11.11	0.001	7.579	2.303	24.938
Extrathyroidal extension (yes versus no)	0.278	9.668	0.002	2.370	1.376	4.082
Constant	1.430	0.027	0.101	0.057		

**Table 3:** Relationship between tumor diameter and CLNMs.

Tumor size (cm)	CLN		Univariate analysis	
	Positive	Negative	*χ* ^2^	*p*
<0.25	2	24	48.674	<0.001
0.25–0.49	22	75		
0.50–0.74	48	111		
0.75–0.99	25	30		
≥1	110	96		

**Table 4:** Risk factors in PTC patients with tumor size <0.25 cm and ≥0.25 cm.

	Tumor size <0.25 cm (26)		Tumor size ≥0.25 cm (517)	
Risk factors	CLN			
	Positive	Negative	Positive	Negative
Gender				
Female	2	17	142	249
Male	0	7	63	63
Age (years)				
<45	1	9	101	108
≥45	1	15	104	204
Bilaterality				
Yes	2	3	59	70
No	0	21	146	242
Extrathyroidal extension				
Yes	0	0	22	14
No	2	24	183	298
